# Beyond ethanol: wine, biological aging, and the Mediterranean context

**DOI:** 10.3389/ijph.2026.1609910

**Published:** 2026-05-15

**Authors:** Alessandro Medoro, Giovanni Scapagnini, Sergio Davinelli

**Affiliations:** Department of Medicine and Health Sciences “V. Tiberio”, University of Molise, Campobasso, Italy

**Keywords:** biological aging, ethanol, Mediterranean diet, polyphenols, wine consumption

Dear Editors,

We read with great interest the article by Esposito and colleagues reporting an association between moderate wine consumption and delayed biological aging in men from the Moli-sani cohort, and we wish to offer some considerations on the methodological and biological implications of these findings [1].

Most studies on alcohol and health outcomes, including biological aging, have considered ethanol as a simple, dose-dependent exposure [2, 3]. This approach does not fully account for the compositional complexity of alcoholic beverages, the biological activity of non-ethanolic compounds, or the dietary context in which alcohol is usually consumed, particularly in Mediterranean populations. Wine, especially red wine, contains hundreds of bioactive compounds beyond ethanol, including flavonoids, phenolic acids, and stilbenes, whose effects on inflammation, oxidative stress, and metabolic function can be independent of ethanol and, in part, may counteract its toxic actions [4]. Likewise, dose, beverage type, pattern of intake, and dietary context are rarely examined together in epidemiological research [5].

The study by Esposito and colleagues in the Moli-sani cohort provides important new evidence on this issue. In 22,495 participants from a large population-based cohort in Southern Italy, biological age was estimated using a deep neural network (DNN) trained on 36 circulating biomarkers covering glucose homeostasis, lipid metabolism, hepatic and renal function, hematological indices, and systemic inflammation. The difference between biological age and chronological age (Δage) was the primary outcome. The key finding is the clear separation between wine and total ethanol as exposures. Overall, ethanol intake was neutral at moderate levels and associated with faster biological aging at higher doses. Conversely, moderate wine consumption, defined according to a traditional Mediterranean diet score, was independently associated with lower Δage in men. The dose-response was J-shaped, with the lowest Δage around 170 mL per day. No significant association was observed in women. If the effect was due only to ethanol, wine and total alcohol intake should have shown similar associations. However, the results showed a clear difference [1].

Blood-based biomarker clocks are more sensitive to dietary and lifestyle changes than DNA methylation-based epigenetic clocks because circulating markers show the current functional state of multiple organ systems [6]. This makes the Moli-sani DNN clock particularly appropriate for detecting the influence of dietary patterns on biological aging trajectories. One important limitation is that the neural network was trained with chronological age as the target. As a result, Δage measures deviation from the average aging trajectory of the study population rather than an individual rate of aging. The cross-sectional design limits causal inference, and residual confounding factors from the healthier lifestyle typical of moderate wine drinkers, such as lower BMI, higher physical activity, and greater Mediterranean diet adherence, cannot be fully excluded despite multivariable adjustment [1]. Supporting evidence comes from the Coronary Artery Risk Development in Young Adults (CARDIA) study, where cumulative liquor and total alcohol consumption were associated with accelerated GrimAge, while wine showed no significant association with epigenetic age acceleration. The consistency across two independent cohorts and two different aging measures strengthens the conclusion that wine-specific components, rather than ethanol, contribute to the observed pattern [7]. Several biological effects may explain this pattern, including the anti-inflammatory and antioxidant effects of wine bioactive compounds, together with favorable changes in lipid profiles, endothelial function, and insulin sensitivity [8, 9]. The evidence supporting resveratrol as the compound responsible for these effects remains weak. In the InCHIANTI study, urinary resveratrol metabolites showed no association with all-cause mortality, cardiovascular disease, cancer, or inflammatory markers. At plasma concentrations achievable with moderate wine intake, resveratrol is insufficient to drive significant biological effects in humans [10]. The flavonoids present at higher concentrations in red wine, such as quercetin, catechin, epicatechin, procyanidin dimers B1–B4, and anthocyanins, represent more biologically relevant candidates [11]. These compounds activate the Keap1–Nrf2 pathway, increase antioxidant enzyme expression, including HO-1 and SOD, and suppress NF-κB-driven production of pro-inflammatory cytokines such as TNF-α, IL-6, and IL-1β [12]. These effects are consistent with many of the inflammatory and metabolic biomarkers included in the Moli-sani blood-based aging clock [1].

The absence of a significant association in women deserves attention. Women typically show lower gastric alcohol dehydrogenase activity and reduced first-pass ethanol metabolism compared with men, leading to higher systemic ethanol exposure per unit of wine consumed. This pharmacokinetic difference may partly attenuate potential polyphenol benefits on the composite Δage. In addition, only 9.3% of women in the Moli-sani cohort fell into the Mediterranean-moderate wine consumption category, compared with 22.6% of men, limiting statistical power to detect a J-shaped relationship. Whether the sex difference is mainly biological or partly due to differences in exposure range among women remains to be clarified with dedicated analyses in cohorts that have sufficient numbers of women at different consumption levels [1, 13].

Alcohol exposure cannot be reduced to grams of ethanol per day. Beverage type, pattern of intake, and dietary context all contribute to the associations seen with cardiovascular outcomes, mortality, and biological aging [1, 14]. The Mediterranean Alcohol Drinking Pattern (MADP), developed by Gea and colleagues in the SUN cohort, combines seven dimensions such as moderate quantity, distribution throughout the week, low spirits consumption, preference for wine (especially red), intake during meals, and avoidance of binge drinking. Higher adherence to this pattern was associated with lower all-cause mortality compared with abstainers and those with irregular drinking habits, an effect that persisted independently of its individual components [14]. Consuming wine with meals rich in fiber, unsaturated fats, and plant polyphenols has a clear biological basis. It delays gastric transit, reduces peak blood ethanol levels, and modifies flavonoid absorption and metabolism through matrix interactions in the gut. The dietary matrix is therefore not simply a variable to adjust, but a critical determinant of effective polyphenol exposure [15–17].

Future studies should better address the complexity of this exposure. Longitudinal cohorts with repeated Δage measurements are needed to clarify the direction of the associations. Objective biomarkers such as urinary tartaric acid and quercetin conjugates should complement self-reported intake to reduce measurement error [18]. Modeling drinking behavior as a multidimensional pattern, rather than total ethanol grams, would improve exposure assessment. Sex-stratified analyses with adequate statistical power in the moderate wine consumption range remain essential. Integrating broader exposome data, such as physical activity, dietary quality, psychosocial stress, and circadian rhythms, will help determine whether the benefit linked to moderate wine consumption belongs to the full behavioral complex rather than to any isolated component. The question is not what is in the glass, but the conditions under which it is consumed.
